# Adoptive cell therapy and modulation of the tumour microenvironment: new insights from ASCO 2016

**DOI:** 10.3332/ecancer.2016.ed59

**Published:** 2016-09-01

**Authors:** Leila Khoja, Bishal Gyawali

**Affiliations:** 1 Royal Free Hospital, Pond Street, London NW3 2QG, UK.; 2 Astrazeneca PLC, Cambridgeshire SG8 6HB, UK; 3 Department of Haemato-Oncology, Nobel Hospital, Kathmandu 21034, Nepal

**Keywords:** immunotherapy, adoptive cell therapy, immuno-oncology, chimeric antigen receptor-modified T cells, tumour infiltrating lymphocytes

## Abstract

Immuno-oncology has changed the landscape of cancer treatment in recent years. Immune checkpoint inhibitors (ICI) have shown survival advantage with long term remissions in a variety of cancers. However, there is another approach to harnessing the power of the immune system in combating cancer: the adoptive cell therapy (ACT) strategy. Although ACT is restricted to small specialized centres and has yet to deliver as much success as ICI, some important results were presented at this year’s ASCO meeting. Important lessons have been learned from these studies, including the prospects and challenges ahead. In this editorial, we summarize the important studies on ACT presented at the ASCO 2016 meeting and discuss the way forward.

Because of the remarkable success achieved with immune checkpoint inhibitors (ICI), most of the current immune-oncology research is focused on finding the optimal treatment schedules, combination partners and managing toxicities of these agents. However, another important facet of immuno-oncology with huge potential for clinical benefit is the strategy of Adoptive Cell Therapy (ACT). Although both these strategies act via enhancing the body’s immunity against cancer cells, they differ in one important aspect. While the ICI basically unleashes the already present immune response by blocking the immune-inhibitory molecules or their receptors, ACT may be able to make a tumour more immune responsive by harvesting T cells from the patient, expanding them in-vitro and reinfusing them into the host.

In accordance with the current trend, immunooncology remained the major attraction at the annual meeting of the American Society of Clinical Oncology (ASCO) 2016. This year though, in addition to the numerous abstracts, oral discussions and presentations on ICI in various tumours and in various combinations with small molecules and other antibodies, there were also a number of presentations on ACT. There were 8 oral presentations during the congress, 5 on chimeric antigen receptor-modified T cells (CART) in haematological cancers [1–5], 2 in solid tumours [6–7] and 1 randomized study using tumour-infiltrating lymphocytes (TIL) therapy in melanoma [8] (Table 1).

CART therapy responses in refractory haematological malignancies have been remarkable [9]. All the haematological studies presented at this ASCO meeting also showed impressive complete response (CR) rates in refractory disease. These were early, by day 28, deep and durable with minimal residual disease (negative bone marrows) following treatment. However, relapses could occur post CR and if they did, they seemed to occur within 6 months of treatment. There were other common themes to the studies: preconditioning chemotherapy especially fludarabine was important in getting the T cells to expand and engraft and toxicities could be very severe with treatment-related deaths occurring in at least 2 studies. The most common toxicities were cytokine release syndrome (thought to be the cause of the treatment related deaths) and neurotoxicity (confusion, delirium, aphasia, seizures, all of which completely resolved). The number of T cells infused and cytokine profiles were associated with toxicity as was the disease burden. Toxicities may be mitigated by altering the dose of infused cells depending on tumour burden.

TIL therapy has been trialed primarily in melanoma with some success largely before ICI [10–11]. The TIL therapy trial from the National Institute of Health (NIH) was interesting in that it was a randomized trial between two protocols that NIH has evaluated in several single arm studies [8]. Preconditioning chemotherapy plus or minus total body irradiation (TBI) was given to 101 patients, with 50 and 51 patients in each arm. Responses and outcomes were similar but toxicity increased in the TBI arm with an idiosyncratic side effect of thrombotic microangiopathy leading to the only death in the trial. Overall the NIH has now treated 196 melanoma patients with TIL, 44 CR (23%) have been seen and 42 of these are ongoing 14–140 months post treatment (there is a 62% partial response rate). Single digit numbers of patients had had ICI previously and they seemed to do worse but it is anecdotal.

In solid tumours the outcomes were much less impressive but one approach in particular was interesting. An EBV sensitized cytotoxic T cell was selected from a panel of over 300 GMP grade lines generated when EBV seropositive haematopoietic donor T cells were sensitized with irradiated transformed B-cell lines with EBV 95.8 and matching was done for at least 2/8 HLA alleles and exhibiting EBV-specific cytotoxicity restricted by an HLA allele shared by the patient [6]. To date, ACT has always been autologous making it labor intensive and expensive. Creating an off- the-shelf product would significantly simplify the process and possibly increase the number of eligible patients.

Finally other engineered cells are also being evaluated. A phase Ib/II trial of expanded and activated autologous natural killer (NK) cells with trastuzumab in refractory HER2+ metastatic breast cancer (MBC) [12] poster presentation described a trial designed to increase the antibody-dependent cell-mediated cytotoxicity effect of trastuzumab. Cells were harvested from patients and incubated with irradiated k562-mb15-41BBl cells for 10 days. Eleven patients were treated with trastuzumab (3 weekly up to 8 cycles). Cycle 1 was followed by a NK cell infusion and IL2 1MU/m^2^ x 3 weekly for 6 doses. No responses were seen but 7 of 10 patients had stable disease and 3 patients had a second infusion at Cycle 6 and 2 patients had a third infusion at Cycle 8. NK cells did expand with increased ADCC but these expansions were modest.

Immunophenotyping of cells in tumours may ultimately help in identifying patients that can benefit from immunotherapies. Immunoscore in colorectal cancer [13] has been validated now and there were numerous presentations on biomarkers of response to ICI. Conversely there is also a need for biomarkers of resistance to identify patients who simply do not respond to immunotherapy. The recently proposed ‘Cancer Immunogram’ could serve as a framework to think of effective immunotherapy on an individualized basis [14]. One requirement in this immunogram is immune (T-cell) infiltration. It could therefore be proposed that so called immunologically cold tumours are suitable for ACT.

This was a topic for discussion in an educational session on CART in sarcoma by Seth Pollack of the Fred Hutchinson Centre. Synovial and myxoid round cell sarcomas are homogenous for NYESO positivity (a cancer testes antigen) but apart from some early data on TIL in synovial sarcoma from NIH, immunotherapy (including ICI) has failed to impress. Biopsies were done in patients at baseline and during treatment. These tumours are described as immunologically cold tumours: very few T cells are seen to infiltrate the tumour and these have low PD1 staining in addition to low PDL1 staining in the tumour. Large numbers of CD168+ (M2 macrophages) are seen but MHC I and II expression is low. A trial was started of IFNg and preconditioning chemotherapy followed by an engineered T cell to NYESO infusion and low dose Il2. There was 1 partial response but also 1 death from myositis thought to be cyclophosphamide related and the study has halted. The concept of modulating the microenvironment to be more immunogenic is also being tested with the use of the gp100 TCR construct produced by immunocore [15]. The antibody to IMCgp100 is coupled to a TCR thus attracting the T cells into the tumour once they bind to tumour cells. A three arm study is underway; alone, in combination with durvulumab (PDL1) or in combination with combination durvulumab and tremelimumab (NCT02535078).

So where does ACT fit in the current landscape of cancer treatment? In haematological disease it looks as if it has a place particularly in refractory disease and especially if the toxicity can be better mitigated. It is conceivable that if toxicity is improved upon it may find a place earlier in treatment paradigms. In solid tumours the responses are poor so that combinations possibly with ICI might be an area to explore. Alternately engineered T cells constructed so as to release stimulatory cytokines such as IL-12 or not express PD-1 could have greater efficacy. TIL therapy at least in melanoma looks more promising and will be a continued area for research possibly in combination with other agents like anti-angiogenics or ICI to increase efficacy further. One drawback of all these ACT approaches, toxicity aside, is the autologous nature of the constructs. T-cells must be harvested and then reinfused post expansion. Treatment with ACT only takes place in specialized centres although the number of such centres worldwide is increasing. Commercialization of this therapy will require an off-the-shelf product that can be used in a wide population of patients. New molecular techniques such as CRISPR/Cas 9 should facilitate this and indeed the first trial in humans is planned for the end of the year [16].

## The way forward

Single agent ICIs, especially anit-PD-1 antibodies, carry relatively low toxicity profiles when compared to chemotherapy. The desire to build upon single agent activity has seen a multitude of different combination strategies explored in clinical trials. However, even with combination immunotherapies, nearly half of the patients do not respond. Without validated predictive biomarkers to select patients for either single agent or a specific combination it will be challenging to determine which patients respond to particular therapeutic strategies or indeed if all patients will respond to immunotherapy given the right combination. Furthermore, the combinatorial approach with at least 2 agents will increase toxicity compromising the applicability to wide patient populations.

ACT can cause life threatening toxicities but reported efficacies, particularly in refractive haematological malignancies, is impressive. Thus, reducing the toxicities of ACT should be the prime focus of future research. Without mitigating the associated toxicities, ACT cannot gain wider application notwithstanding survival results in phase 3 trials. In solid tumours, the lack of a predictive biomarker further hinders the wider use of this therapy although immunologically cold tumours may be particularly suited to the ACT approach and could be hypothesized to be resistant to ICI therapy. Response to ACT post ICI is unknown at the current time as the majority of available ACT data is from patients pre-ICI development.

## Conclusion

Although significant challenges lie ahead especially in solid tumours, ACT might find its place in the treatment paradigms of a variety of tumours if and when the two major problems associated with ACT are mitigated: the logistics in administering the ACT therapy and the seriousness of the toxicities.

## Conflicts of Interest

Dr Khoja works as a research physician for Astrazeneca but this work is independent of Astrazeneca. Dr Gyawali has no conflicts of interest to declare.

## Figures and Tables

**Table 1.** Important studies of ACT presented at ASCO 2016.

	Study	Centre	Antigen/vector/CART	Trial detail	Efficacy	Safety
1	Rate of durable complete response in ALL, NHL, and CLL after immunotherapy with optimized lymphodepletion and defined composition CD19 CAR-T cells [1].	Fred Hutchinson Cancer Center	CD-19, lentivirus, 2^nd^ generation (41BB) CD4:CD8 1:1 ratio (Autologous)	N = 90 overall **ALL n = 36**: Heavily pre-treated (median 3(1–11) Lymphodepletion (cy alone or CY/flu) then CART, fludarabine needed for optimal cell expansion and optimal survival. Three doses of CART used, MTD 2 × 10^6^ Outpatient treatment in 72%	CR: 32/34 (94%)	2 deaths Toxicity related to number of CART infused and bone marrow blast count Risk adapted dosing was proposed based on marrow blast percentage so that if patients had ≥ 20% BM blasts then they received ≤ 2x10^5^/kg but if they had <20% BM Blasts the cell infusion was ≤2 × 10^6^/kg Toxicity seen in n = 19: CRS G0-2:74%, G3-4: 21%, G5 5% 26% neurotoxicity Median days in hospital was 6 days
				**NHL n = 41** Median 4 (1–11) prev treatments. MTD 2 × 10^6^ Fludarabine needed Outpatient treatment in 68%	CR 33% overall (50% with flu and higher dose level, ORR 80%)	2 deaths Toxicity seen in n = 20 CRS G0-2 90%, G3-4 10%, G5:0. Neurotoxicity was seen in 10% Median days in hospital was 5 days
				**CLL n = 13** 77% treated as outpatient Poor prognosis disease Cy/flu needed Irbutinib refractory/intolerant	Cy/flu: ORR 10/11 (91%) CR 5/11 (45%) BM negative 10/11 (91%)	Toxicity seen in n = 13 CRS G0-2 77%, G3-4 23%, G5 0 Neurotoxicity was seen in 23% Median days in hospital was 8 days
2	Sustained remissions with CD19-specific chimeric antigen receptor (CAR)-modified T cells in children with relapsed/refractory ALL[3]	Children’s hospital Philadelphia	CD-19, lentivirus, 2^nd^ generation (41BB)	Chemotherapy (at discretion whilst CART made) then lymphodepletion Cyc/flu	Cr 56/60 (93%) 12 month RFS 60%	CRS mimics macrophage activation syndrome/haemophilic lympho histocytosis and has the same cytokine profile; IFNg, IL13 and MIP 13a. Toxicity also associated with hepatomegaly and splenomegaly. A predictive model was developed using these cytokines and disease burden.
3	Efficacy of humanized CD19-targeted chimeric antigen receptor (CAR)-modified T cells in children with relapsed ALL [4].	Children’s hospital Philadelphia	CD-19, lentivirus, 2^nd^ generation (41BB) Humanised scFV (as opposed to murine) to test that IMMUNOGENICITY contributes to poor persistence.	Previously treated with CART, PR/PD or relapse, B cell recovery seen N = 10	6/10 : CR but 2 of these relapsed, some had no response to previous murine CART	CRS: no G4. Toxicity correlated with T cell proliferation and disease burden
4	Randomized, phase II dose optimization study of chimeric antigen receptor (CAR) modified T cells directed against CD19 in patients (pts) with relapsed, refractory (R/R) CLL [2]	Pennsylvania-Abraham’s cancer centre	CD-19, lentivirus, 2^nd^ generation (41BB)	Two (equal efficacy seen previously) arms of n = 12: 1–5 × 10^7^ vs 1–5 × 10^8^ Additional patients once optimal dose defined. Patients relapsed, refractory (within 2 years of prev tx) adults, at least 2 prev treatments 18% had flu/cyc (others a mixture) n=17 treated at optimal dose (5 × 10^8^), relapses happen at 3 months,	CR: 6/24 (25%,) PR: 4/24 (17%) ORR: 42% Higher dose had higher ORR N=17 ORR 53%, CR 35% many >2yr	Toxicity seen included tumour lysis syndrome B cell aplasia, hyogammaglobulinaema CRS was seen in 55%/54% no relation to dose
5	Anti-CD19 chimeric antigen receptor T cells preceded by low-dose chemotherapy to induce remissions of advanced lymphoma [5]	NCI	DNA encoding anti-CD-19 CAR is ligated into MSGV gammaretroviral backbone. Unselected peripheral PBMCs are transduced to create CD19 CART	N=22 19 DLBCL, 2 follicular, 1 mantle cell Flu 30mg/m^2^ and cyc 300mg or 500mg/m^2^ given preinfusion 1 × 10^6^/kg, 2 × 10^6^/kg or 6 × 10^6^/kg	ORR 73% Cr 55% PR 18% DLBCL CR 47% PR 21%	55% had G3/4 neurotoxicity 18% had G3/4 hypotension IL-10, Il-15 levels were elevated in patients who had CR/PR Granzyme B, IL-10, IL-15 levels were elevated in patients experiencing neurotoxicity
6	Treatment of EBV+ nasopharyngeal carcinoma with banked EBV-specific cytotoxic T cells [6]	MSK	EBC CTL selected from >300 GMP grade lines generated from EBV seropositive hematopoietic transplant donors (T cells sensitised with irradiated transformed B-cell lines with EBV 95.8 and matching was done for at least 2/8 HLA alleles and exhibiting EBV-specific cytotoxicity restricted by an HLA allele shared by the patient.	3 weekly doses given of 1–2 × 10^6^/kg for median 2 cycles (1-4) N = 14	CR:1 for 21.2 months PR:2 for 3.5 and 6.7 months SD:1 for 7.4 months 11/14 still alive at median 18.1 m (3-48.4)	None reported
7	Anti-mesothelin chimeric antigen receptor T cells in patients with epithelial ovarian cancer [7]	University of Pennsylvania	Anti-mesothelin: mouse mAb SS1 derived. 2^nd^ generation (41BB co stimulatory). Lentivirus vector. (CARTmeso is immunogenic) (Humanised CARTmeso will enter phase I trial)	Single CART-meso infusion (1–3 × 10^7^/m^2^ or 1–3 × 10^8^/m^2^) alone or post lymphodepletion with cyc 1.5g/m^2^	Poor CART expansion and persistence but did traffic to tumour sites. SD: 6/6 patients	No CRS seen
8	A randomized, prospective evaluation comparing intensity of lymphodepletion prior to adoptive transfer of tumour infiltrating lymphocytes for patients with metastatic melanoma [8]	NIH	Tumour infiltrating lymphocytes (TIL)	N = 101 Randomised 1:1 study of preconditioning chemotherapy (NMA) plus or minus whole body irradiation (1200) (TBI) followed by TIL treatment and high dose IL-2	CR n = 12 (24%) in each arm-ongoing bar one. Median survival (months) OS NMA 36.6 TBI 38.2 PFS NMA 7.5 TBI 9.6	Arms were balanced for site of TIL harvest and doses of IL-2 administered (median 5/6 range 4–7). Toxicity increased in the TBI arm; longer neutropenic periods, thrombotic micro-angiopathy affecting the kidneys predominantly and increased weight loss. Two deaths in TBI arm (one from toxicity one other cause)

ALL: Acute Lymphoblastic Leukaemia, NHL: Non-Hodgkin’s Lymphoma, CR: Complete Response Rate, ORR: Objective Response Rate, CRS: cytokine release syndrome
